# Effects of Weed Control Practices on Plant Diversity in a Homogenous Olive-Dominated Landscape (South-East of Italy)

**DOI:** 10.3390/plants10061090

**Published:** 2021-05-29

**Authors:** Massimo Terzi, Emanuele Barca, Eugenio Cazzato, Francesco Saverio D’Amico, Cesare Lasorella, Mariano Fracchiolla

**Affiliations:** 1 Institute of Biosciences and Bioresources, National Research Council, Via Amendola 165/A, 70126 Bari, Italy; massimo.terzi@ibbr.cnr.it; 2 Water Research Institute, National Research Council, Via F. De Blasio 5, 70132 Bari, Italy; emanuele.barca@ba.irsa.cnr.it; 3 Department of Agricultural and Environmental Science, University of Bari, Via Orabona 4, 70126 Bari, Italy; cesare.lasorella@uniba.it (C.L.); mariano.fracchiolla@uniba.it (M.F.); 4 Department of Biology, University of Bari, Via Orabona 4, 70126 Bari, Italy; francescosaverio.damico@uniba.it

**Keywords:** biodiversity, chemical herbicides, Mediterranean, mowing, olive groves, tillage, weeds

## Abstract

Olive groves represent an important economic, agro-ecological, and cultural resource in the Mediterranean Basin. Weed management plays a fundamental role in their sustainable management. The aim of this work was to characterize and assess the plant diversity associated with different weed control practices, in a homogeneous olive-dominated landscape in the South-East of Italy. Sixty-five vegetation plots were sampled in orchards treated with different weed control practices: mowing, tillage, and use of chemical herbicides. The multi-response permutation procedure was used to test the hypothesis of no difference among the treatments. The relationships between plots were visualized by means of non-metric multidimensional scaling ordination. A generalized linear mixed model was used to analyze the relationships between weed control practices and life forms, chorotypes, and diversity indexes. The results showed that the three weed control practices determined slightly different plant communities. Chemically weeded orchards showed an impoverished floristic composition and the lowest diversity, whereas mowing and tillage yielded similar values. These latter two treatments differed for the percentages of hemicryptophytes and therophytes. Moreover, different from other studies, we did not find plant species of particular concern for biodiversity conservation. We hypothesize that this result is due to the monotonous structure of the agro-landscape we investigated, where natural elements are almost lacking. From this point of view, a correct management of agro-districts should consider both the agronomic practices at the level of the individual olive groves and the structure of the agro-landscape.

## 1. Introduction

The olive tree (*Olea europaea* L.) is an emblematic plant of the agricultural landscape of the Mediterranean Basin, where it was cultivated by the most ancient civilizations of the area [1]. Olive tree cultivations came to dominate many rural landscapes, and, nowadays, the countries bordering the Mediterranean Basin include over 90% of the total area worldwide, with the largest harvest areas being in Spain, Tunisia, and Italy [2]. As a consequence, the olive tree represents an important economic, agro-ecological, and cultural resource for this area [3,4]. It has traditionally been grown in extensive dry farming, characterized by low densities and poorly mechanized. Since the 1970s, overall, the adoption of irrigation and other mechanized management practices has deeply changed olive-growing systems. The intensification of external inputs (e.g., herbicides), the increase in tree density, as well as other technical improvements have increased productivity but have also produced new threats to biodiversity [5].

Biodiversity provides many ecological services in agro-ecosystems (beyond the production of food), such as recycling of nutrients, regulation of microclimate and local hydrological processes, suppression of undesirable organisms, and detoxification of noxious chemicals [6]. The influence of weed management on agro-ecosystem biodiversity has been demonstrated for several orchards and herbaceous crops, see for example [7,8,9,10]. Regarding olive groves, different weed control practices (WCPs) have been compared in some European Mediterranean countries; it has been shown that soil management, as well as having effects on soil quality, erosion, fertility, and carbon stocks [11,12,13], exerts a strong effect on the diversity of some taxonomic groups, such as vascular plants, reptiles, birds, and arthropods [8,14,15,16,17,18,19]. The ecological importance of olive groves for biodiversity conservation has been highlighted by several authors, for example by [20,21,22,23,24]. Moreover, under some circumstances, olive groves have been considered as forming “High Nature Value” farmlands [25].

The ecological function of rural landscapes and the promotion of multifunctional agriculture are important topics in agricultural–environmental policy within the European Union [26]. The Common Agricultural Policy post-2020 supports actions among farmers to reach goals such as improved landscape connectivity and supporting farms in High Nature Value areas [26].

The scientific literature agrees that the level of biodiversity is a reliable indication of the ability of an agricultural environment to provide services to the environment and human healthiness [27]. Therefore, effective management of biodiversity of olive groves can have a positive holistic impact on a large and significant area of the Mediterranean Basin.

In Italy, one of the world’s leading countries for olive production, nearly a third of olive tree orchards are concentrated in the Apulia region [28]. In the central part of the region, in some municipalities of the basal belt, olive cultivations can reach more than 90% of the utilized agricultural area. This productive system is composed of a homogeneous olive-dominated landscape, where natural habitats are poorly represented.

The aim of this study was to characterize and assess the plant diversity associated with this important productive district, in relation to the main WCPs used in the area—here broadly classified as tillage (Ti), mechanical mowing (Mo), and chemical herbicides (He). This paper provides answers to the following questions:

- Do different WCPs lead to different weed communities?
- How do WCPs affect plant diversity?
- Finally, what recommendations can be drawn for a sustainable management of the olive orchard agro-environments?

Different from other studies carried out on a few experimental plots concentrated in only one or a few sites, where the agronomic practices can be precisely controlled, the present work loses this precision but gains insights into the variability of weed communities along the entire productive district in relation to the main WCPs used in the area.

## 2. Results

Considering all the analyzed plots, 161 taxa (species and subspecies) belonging to 138 genera were recorded. The mean number of taxa was 15 for chemical herbicides (He), 26 for mowing (Mo), and 27 for tillage (Ti). The most common species, recorded in more than 60% of the relevés, were *Lolium rigidum*, *Hypochaeris achyrophorus*, *Sonchus oleraceus*, *Medicago orbicularis*, and *Erodium malacoides*. All the taxa are widespread in the region and lack any particular interest for biodiversity conservation (except two *Serapias* sp. records). Many taxa occurred in only one or two plots, revealing a large number of accidentals. Few taxa (except accidentals) turned out to occur exclusively in only one WCP (Supplementary Table S1).

Checking for significant differences between weed control practices (WCPs; described in the Material and Methods section), the multiple response permutation procedure (MRPP) indicated significant differences in floristic composition between the groups of plots managed with the different WCPs, both for presence/absence and abundance–dominance data. The T values, however, indicated a stronger separation between groups when considering the species abundance–dominance values (Table 1).

The pairwise comparisons between the three groups showed the He group to be well differentiated from the others. Therefore, a first main distinction is between Ti and Mo from one side and He on the other.

**Figure 1:**
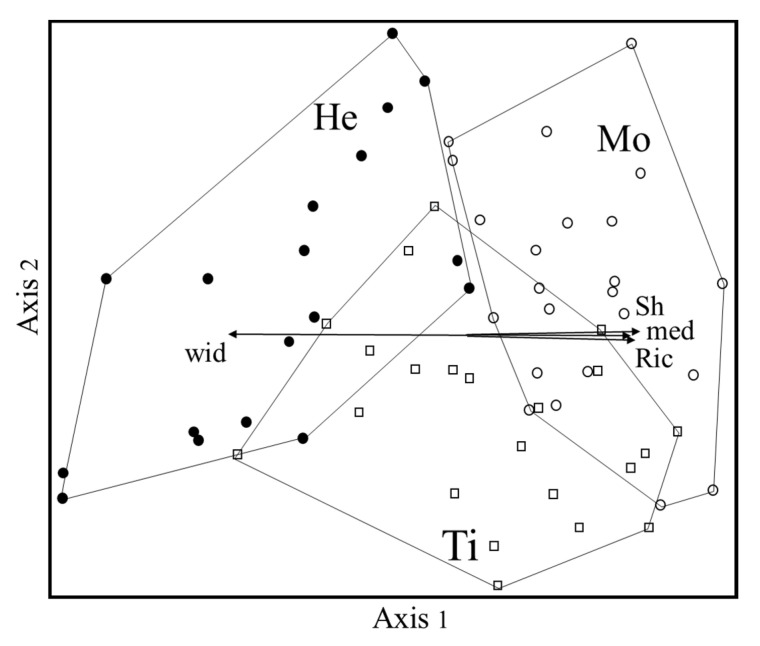
NMS ordination diagram. Ti = Tillage (open squares); He = Chemical herbicides (filled circle); Mo = Mowing (empty circles); med = Mediterranean taxa; wid = Taxa with wide distribution; Ric = Richness; Sh = Shannon index.

The non-metric multidimensional scaling ordination (NMS, Figure 1), carried out to visualize the floristic relationships among the three WCPs, resulted in a three-axes solution, with a final stress of 17.6. The first two axes account for most of the variance, 43.4% and 21%, respectively (the third axis, not shown, explained 10.6%).

In the ordination diagram, the Mo and He groups are clearly separated by Axis 1, irrespective of the altitude of the vegetation plots. The Ti group—which is more differentiated by Axis 2—partially overlaps the others, especially mowing.

Axis 1 turned out to be positively correlated with the Mediterranean chorotype and with richness and Shannon indexes. Group He has a higher percentage of taxa with wide distribution and lower diversity with respect to Mo.

After a correlation analysis, the treatments were selected as predictors for a generalized linear mixed model (GLMM) model application. This analysis showed also that tree density does not significantly affect the model. As expected, steno- and eury-Mediterranean taxa prevail in the three groups, although they showed lower percentages in orchards managed with chemical herbicides. Moreover, species with wide distribution (Turanian–Mediterranean) are more represented in chemically weeded orchards (Supplementary Table S2, Table 2).

Group He has lower diversity (richness and Shannon indexes) with respect to Mo and Ti. Therophytes are the dominant life form everywhere (Supplementary Table S2). Ti and Mo groups, however, differ in the percentages of hemicryptophytes and therophytes, being higher and lower, respectively, in the Mo group (Table 2).

Regarding the analysis of indicator species, which was carried out to identify species associated with the WCPs, the three groups and their combinations turned out to be associated with few indicator species (IndSp), often with low indicator values (IndVals) (Supplementary Table S1). Considering IndSp with IndVal > 25, three taxa were associated to Ti (*Glebionis segetum*, *Avena sterilis*, *Melilotus sulcatus*), five to Mo (*Crepis bursifolia*, *Galium murale*, *Astragalus hamosus*, *Geranium molle*, *Rostraria cristata*), and only two to He (*Cardamine hirsuta*, *Capsella rubella*). In addition, eight taxa avoid the orchards treated with chemical herbicide (*Urospermum picroides*, *Crepis sancta* subsp. *nemausensis*, *Avena barbata*, *Hordeum murinum* subsp. *leporinum*, *Medicago truncatula*, *Anisantha madritensis*, *Sherardia arvensis*, and *Lotus ornithopodioides*) along with one in the Mo group (*Senecio vulgaris* subsp. *vulgaris*). Seven IndSp yielded the highest IndVal for the trivial partition with all the vegetation plots in only one group. Most of the IndSp are diagnostic species of the *Chenopodietea* class and *Brometalia rubenti-tectorum* order, which include the Mediterranean ruderal vegetation of anthropogenic habitats [29].

## 3. Discussion

The results show that different WCPs determined slightly different plant communities, although the differences are clearer when considering the cover abundance of taxa rather than their presence/absence. Orchards managed with chemical herbicides turned out to be well distinguished from the others and characterized by an impoverished floristic composition, with few IndSp, and lower diversity. *Lolium rigidum* and *Erigeron canadensis* occur with high frequency in the three groups, but in He they represent the main dominant taxa. It is likely that these are glyphosate-resistant biotypes [30,31], as their presence was ascertained in the study area in which this herbicide is widely used [32].

It is well known that the use of chemical herbicides reduces biodiversity in olive orchards as well as in other perennial crops [4,8,9,17,19,33,34]. Our findings confirm these data and show that the use of only chemical herbicides for weed control does not represent a rational strategy.

In the past half-century, there has been a substantial divergence of intentions between agronomy and ecologists [35]: the former interested in minimizing yield losses, the latter in preserving ecosystem functionality. Subsequent considerations regarding sustainability and new scientific acquisitions rely on a common set of ecological and agronomic principles that bring together many fields of research [36]. More precisely, it is now a common belief that increasing or maintaining high levels of biodiversity is a crucial goal in the sustainable management of agro-environments [35,36].

First of all, the more diverse a weed community is, the less competitive it is, because the phenotypic differentiation among species contrasts the impoverishing of resources [36].

Regarding pest management, ecological bases of interactions between weeds and other organisms (including beneficial) are debated, for example, by Norris and Kogan [37], Norris [38], and Barberi et al. [27]. Moreover, the fauna community is strongly linked to natural flora, which provides biomass, pollen, cover, and reproduction sites. For instance, Marshall et al. [39] reported that at least 15 genera of weeds are very important, important, or present in the diet of birds of agricultural lands and highlighted the strong relationship between insects and weeds.

Another important issue is the economic subsistence of companies and its link with biodiversity. An interesting interpretation is reported by Gerowit et al. [40]; the multifunctional performance of agriculture is commonly used as an important factor to argue about the transfer of public money into agriculture. If farmers are required to also produce ecological goods, they need to produce, first of all, biodiversity, which is the main indicator of the ecological performance of agro-ecosystems [6]. In this type of approach, weed diversity can be used to detect environmentally friendly cropping systems [40].

Obviously, density or total biomass must be reduced in the period of the year, such as spring and summer, in which weeds can compete for minerals and water, but weed control strategies must ensure the improvement or the maintenance of a high level of diversity.

Integrated weed management is “the application of numerous alternative weed control measures, which include cultural, genetic, mechanical, biological, and chemical means of weed control” [41]. This approach to weed control has been reported to improve biodiversity [42,43]. We show that tillage and mowing positively affect biodiversity and thus they can be highly recommended, unlike the chemical management of weeds.

From a properly ecological point of view, our study shows that the percentages of therophytes were higher in Ti, where soil tillage brought back vegetation toward the initial stages of the succession, whereas hemicryptophytes, which take advantage in undisturbed soils, as already observed by other authors [7,15], were higher in the Mo group.

Different from other studies that reported a higher biodiversity for mowing [15,44], our results showed that Ti and Mo yielded similar values of the diversity indexes. These different findings could be related to other variables, such as the landscape structure (and function).

Even if our study area was highly homogeneous, it was characterized by a myriad of smallholder farms, among which a great variability of management techniques is observed, according to the choices and needs of different owners (see description in Material and Methods section). Therefore, different control techniques and different times of weed removal determine different ecological conditions.

It is reasonable to hypothesize that this extreme fragmentation, not of the land use but of soil management techniques and timing, allows a consistent species flow between neighboring fields, and few taxa were found to be exclusive to just one of the treatments.

Simoes et al. [15] showed that mowing and tillage determine different permeabilities to species flow: Ti favors pioneer annual species, typical of highly disturbed environments, whereas Mo is more permeable to species of “ecologically diverse communities, with a high natural value”. However, in the olive production district that we have considered, natural habitats are almost completely lacking. Therefore, the indicator species associated with Mo and Ti belong almost only to ruderal syntaxa, such as *Chenopodietea* (*Brometalia rubenti-tectorum*), *Sisymbrietea*, and *Papaveretea rhoeadis* (Supplementary Table S1).

In addition, we did not find taxa of particular concern for biodiversity conservation (i.e., endangered, rare, or endemic taxa). However, this is not always true, as many other studies have stated the importance of olive groves for the conservation of biodiversity [16,22,25,44,45,46,47]. Actually, the lack of endangered or rare plant taxa in our study area was somehow expected due to the monotonous and specialized olive-growing agro-landscape and almost a total lack of natural elements, such as forest remnants. This monotonous landscape thus seems unsuitable to host a wide range of wild species. On the contrary, if the landscape contains suitable natural habitats, then olive orchards can also be used by wild species. Most of the endemic and endangered taxa recorded by Radić Lakoš et al. [44] for the olive orchards near Šibenik (Croatia) are ingressive from neighboring semi-natural grasslands of the *Scorzoneretalia villosae* (i.e., the Amphiadriatic order including dry steppic, submediterranean pastures [48,49]). Carpio et al. [19] have demonstrated that natural elements, orchard hedgerows in particular, have a positive effect on weed communities and the soil seed bank. However, those authors also observed a low relationship between the seed bank and the weed community. The importance of the landscape structure for weed richness was also highlighted by Gabriel et al. [50] who, however, showed this relationship for the weed community of an arable field.

The importance of the agro-landscape structure, and in particular the presence of forest patches, has also been highlighted for other taxonomic groups, such as frugivorous birds and bats [51,52,53,54,55]. It has been shown that forest patches are also important for maintaining soil biodiversity [17] and that agro-landscape complexity (i.e., presence of different land-cover classes) positively affects arthropod richness [18].

The given explanation of those different results is also supported by the work of Allen et al. [56] who found in the traditional olive cultivation of Crete (Greece) many herbaceous species widespread in a variety of ruderal habitats, such as road verges, and hypothesized that an intensification of cultivation would lead to a decrease in diversity of both ground flora and insects, birds, and mammal fauna.

In accordance with some authors [53,55,57], our results suggest that the biodiversity of olive groves depends on both local management practices and the agro-landscape structure. In other words, the importance of olive groves as a habitat suitable for rare taxa is greatly reduced by the homogeneity of the agro-landscape and by the lack of natural elements (e.g., semi-natural grasslands, woodlands). Therefore, proper management of agro-districts should consider both practices at the level of individual olive groves and the structure of the agro-landscape, as heterogeneity and the presence of natural elements can improve biodiversity [50].

## 4. Material and Methods

### 4.1. Study Area

**Figure 2:**
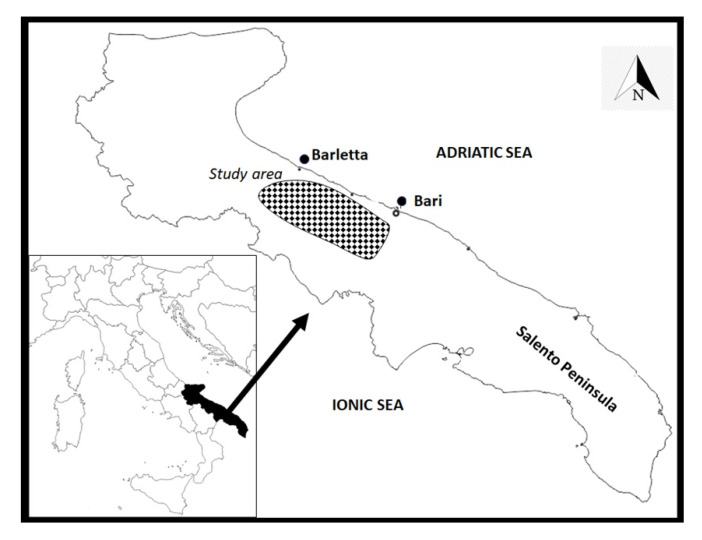
Map of the study area (Apulia region, South-East of Italy). The map was created from templates obtained from: https://d-maps.com/carte.php?num_car=2329&lang=it (accessed on 28 May 2020) and https://d-maps.com/carte.php?num_car=7965&lang=it (accessed on 28 May 2020).

The study area consists of one of the most important productive olive districts of the Apulia region, situated between the cities of Bari and Barletta (Figure 2), and extends from the coast toward the hinterland for nearly 15–20 km, with an altitudinal gradient ranging from a few dozen meters up to 350 m above sea level. The geological substrate is fairly homogeneous and consists of limestone and calcarenites.

According to Rivas-Martínez et al. [58], the bioclimate is Mediterranean pluvioseasonal-oceanic, with a dry ombrotype. A main altitudinal gradient goes from the coast, where the thermotype is thermomediterranean (e.g., Bari, Barletta), toward the hinterland, where it becomes mesomediterranean (e.g., Corato). A second weaker gradient of continentality (slightly higher in the north-western part of the study area) is parallel to the coast. There is quite a homogeneous olive-dominated landscape with orchards that can be defined as intensified traditional in terms of the use of external inputs (e.g., mechanization, chemicals, water). According to the description made by Pienkowski and Beaufoy [4] for olive trees in the EU, they are an intermediate type of olive orchard between “low-input traditional plantations and scattered trees, […] which are managed with few or no chemical inputs” and “intensive modern plantations of smaller tree varieties, planted at high densities”. The area is also characterized by an extreme fragmentation of the land ownership (80% of farms do not exceed 3 ha, as reported by the Italian agricultural census of 2010).

In the context described above, several olive groves were chosen in which weeds were managed by farmers with different strategies (WCPs) that were classified into three broad categories: (1) chemical herbicides (He), including various types of treatments, although foliar ones (glyphosate) are the most commonly used in the area; (2) mowing (Mo), i.e., mechanical shredding of the aboveground portion of weeds without any soil disturbance and leaving residues covering the soil; (3) soil tillage (Ti), with various techniques, although the most common is to use a shallow (at least 20–25 cm) disk or chisel plowing, operating without the inversion of soil, i.e., only stirring it.

Information about WCPs was obtained through interviews with local farmers or based on our knowledge, particularly in farms for which we knew the soil management practices or in which there was full evidence of the techniques used.

Cases where an integrated management was evident, i.e., conducted using multiple techniques, were excluded. The permanent or semi-permanent vegetation cover with cover crops has not been taken into consideration as this practice is rare in the investigated area.

### 4.2. Vegetation Sampling

The sampling of weed vegetation was spatially stratified in order to include sample plots along the two main bioclimatic gradients and further stratified according to the three WCPs. In all, in three years (2016–2017–2018), we selected 65 olive orchards for which we were able to obtain information on how they had been treated. Nearly half of the selected sites were at an altitude lower than 200 m a.s.l. (8 He, 13 Mo, and 12 Ti) and another half at higher altitudes, up to 350 m (10 He, 10 Mo, and 12 Ti).

Weed vegetation was sampled by using the phytosociological method [59]. For each selected olive orchard, a plot with rectangular shape (5 × 3 m or 2 × 7.5 m) was surveyed, almost the same size as that proposed by Chytrý and Otýpková [60] for synanthropic herbaceous vegetation. They were arranged with their longest axis falling parallel to the orchard inter-row so that the olive trunks and their tree circles were excluded. Here, in fact, weed vegetation is often affected by practices other than WCPs (e.g., fertilization). Relevés were located where vegetation cover was at least 60% and at least 4 m away from the orchard borders. Vegetation was sampled between April and the beginning of May, which is immediately before the first spring weed control made during the vegetative growth of olive trees, when the effects of different management practices are clearer. For every relevé, the complete list of vascular plant taxa was recorded. Importance values of taxa were estimated by using the Braun-Blanquet scale [61]; however, these values were replaced by the ordinal scale before the statistical analysis, as proposed by Lepš and Hadincová [62]. Life forms and chorological spectra weighed by taxon scores were calculated for every relevé, on the basis of information provided by Pignatti et al. [63] for Italian flora. Based on the list of diagnostic species provided by Mucina et al. [49] (see also: https://www.synbiosys.alterra.nl/evc/, accessed on 28 March 2020) for European vegetation types, taxa reported in Supplementary Table S1 were associated with one or more syntaxa. The taxonomic nomenclature follows the Euro+Med PlantBase [64].

The floristic diversity of relevés was estimated through the richness and Shannon indexes [65]. Species were considered of conservation interest if they were included in regional [66,67], national [68,69], or European [70] red lists of plants, if their distribution was limited to a restricted area (endemic taxa) [63], or if they have been indicated as rare in the region [1].

### 4.3. Statistical Treatments of Data

The multi-response permutation procedure (MRPP) [71] was used to test the hypothesis of no difference among the three WCP groups of relevés. MRPP tests were run on both presence/absence and abundance–dominance data. The average distances within the three groups were calculated with the Bray–Curtis coefficient. The statistic A expresses the heterogeneity within groups, with A being between 0 (heterogeneity equals expectation by chance) and 1 (all the plots are identical). The test statistic T describes the separation between groups: the stronger the separation, the more negative it is [65,72].

The relevés were ordinated through non-metric multidimensional scaling (NMS) by using the Bray–Curtis coefficient as a dissimilarity measure. NMS was run using the “slow and thorough” autopilot mode in Pc-Ord 6.22 [72]. In the ordination diagram, life forms and chorological spectra weighed by taxon scores were plotted as vectors on a joint plot, with a cut-off threshold of R = 0.3. The altitude of the sampled plots, here used as a proxy of bioclimatic conditions, and richness and Shannon indexes were also passively projected into the ordination diagram.

A generalized linear mixed model (GLMM) was used to analyze the relationships between WCPs and life forms, chorotypes, and richness and Shannon indexes. The GLMM, performed through the R package glmmTMB [73], was designed to overcome the constraints of the classic linear multivariate model, namely the Gaussianity and the independence of the residuals. The number of models processed were 17 but only models with significant outcomes (9) were reported. Richness, Shannon index, Therophytes, Hemicryptophytes, Fabaceae, wide distribution, steno-Mediterranean, Eury-Mediterranean, and Mediterranean-Turanian taxa were the response variables. During the feature selection stage, all the potential predictors were checked to assess their explanatory capability. Elevation proved to be significant only for Boreal and Steno-Mediterranean taxa (as expected), therefore it was discarded from the analysis. The sole covariate used was the factor management. Gaussian distribution was adopted. The GLMM in matrix form can be expressed as follows: Y = Xβ + Zγ + ε, where Y is a N × 1 column vector, the response; X is a N × p matrix of the p predictor variables; β is a p × 1 column vector of the fixed-effects regression coefficients; Z is the N × q design matrix for the q random effects; γ is a q × 1 vector of the random effects; and ε is a N × 1 column vector of the independent residuals, which is part of observed Y that is not explained by the model Xβ + Zγ. Indicator species analysis (ISA) [74] was used to identify the indicator species (IndSp) associated with the three WCP groups and their combinations [75]. For each taxon with more than 2 occurrences, we calculated the indicator value index (IndVal) for the three WCP groups and their combinations. Each taxon was thus associated with the group or combination of groups for which its IndVal yielded the highest value. A randomization (Monte Carlo) test with 10,000 permutations was used to evaluate the statistical significance (*p* < 0.05) of the IndVal. Indicator species were also calculated for the trivial partition containing all the vegetation plots in only one group [74]. MRPP, NMS, and ISA were performed using PCORD, version 6.22 [72].

## 5. Conclusions

This study shows that different weed control practices determine different olive orchard weed communities. A lower plant diversity was associated with orchards treated by chemical herbicides, as stated by many other authors, whereas no appreciable differences of plant diversity were observed between mowing and tillage. In addition, plant taxa of particular conservation concern were lacking in all the treatments, except two *Serapias* sp. records, which could be of a certain conservation interest as reported by Wagensommer et al. [76]. We interpreted these findings assuming that diversity is affected by processes acting at multiple spatial scales, such as local management techniques on one side and landscape functioning (e.g., species flow), on the other. In particular, homogeneous olive-dominated landscapes, without natural elements, such as hedgerows, woods, or semi-natural grasslands, lose importance for biodiversity. In a nutshell, when lacking natural (source) habitats, olive orchards cannot represent (sink) suitable habitats for wild species. Comparing our results with other studies allowed us to hypothesize that orchards managed by mowing could be more permeable to rare and endangered species than those treated by tillage. Overall, our results suggest that the relationships among weeds, weed management, and agro-ecosystem services should be related to the agro-landscapes context in the debate about the multifunctional role of Mediterranean olive groves.

## Figures and Tables

**Table 1:** Results from multi-response permutation procedure (MRPP) tests of no differences among weed communities—run on both presence/absence (P/A) and abundance–dominance (AD) data—and pairwise comparisons between soil tillage (Ti), mowing (Mo), and chemical herbicides (He).

		T	A	*p*-Values
Presence–Absence (P/A)		−12.5	0.16	≤0.001
P/A:	Ti vs. He	−11.07	0.16	≤0.001
P/A:	Ti vs. Mo	−4.61	0.06	≤0.001
P/A:	Mo vs. He	−10.77	0.15	≤0.001
Abundance–Dominance (AD)		−15.83	0.22	≤0.001
AD:	Ti vs. He	−12.64	0.21	≤0.001
AD:	Ti vs. Mo	−5.81	0.08	≤0.001
AD:	Mo vs. He	−14.38	0.23	≤0.001

**Table 2:** List of the response variables with significant predictors and *p*-values after GLMM application.

Response Variables	Significant Predictors	*p*-Values
Diversity		
Richness	Chemical herbicides	≤0.001
Shannon	Chemical herbicides	≤0.001
Life forms		
Hemicryptophytes	Mowing	≤0.001
Therophytes	Mowing	0.004
Chorotypes		
Wide-ranging	Chemical herbicides	≤0.001
Steno-Mediterranean	Chemical herbicides	0.0131
Turano-Mediterranean	Chemical herbicides	≤0.001
Eury-Mediterranean	Chemical herbicides	≤0.001

## Data Availability

Data is contained within the article or supplementary material.

## Supplementary Materials

The following are available online at https://www.mdpi.com/article/10.3390/plants10061090/s1, Table S1: Vegetation plots from olive orchards managed with different weed control practices (WCPs): chemical herbicides (He), tillage (Ti), and mowing (Mo). Chen = *Chenopodietea*; Sisy = *Sisymbrietea*; Papav = *Papaveretea rhoeadis*; St-T = *Stipo-Trachynietea distachyae*; Brom = *Brometalia rubenti-tectorum*; Table S2: Chorological and life forms spectra weighed by taxon scores.

## Abbreviations

WCPs: Weed control practices. He: Chemical herbicides. Mo: Mowing. Ti: Soil tillage.

## References

[1] Pignatti S., Guarino R., La Rosa M. (2017). Flora d’Italia.

[2] (2018). Faostat. http://www.fao.org/faostat/en/#data/QC.

[3] Blondel J., Aronson J. (1999). Biology and Wildlife of the Mediterranean Region.

[4] Pienkowski M., Beaufoy G. (2002). The Environmental Impact of Olive Oil Production in the European Union: Practical Options for Improving the Environmental Impact. European Forum on Nature Conservation and Pastoralism. https://ec.europa.eu/environment/agriculture/pdf/oliveoil.pdf.

[5] Fernández-Escobar R., de la Rosa R., Leon L., Gómez J.A., Testi L., Orgaz F., Gil-Ribes J.A., Quesada-Moraga E., Masallem M. (2013). Evolution and sustainability of the olive production systems. Options Méditerranéennes.

[6] Altieri M.A. (1999). The ecological role of biodiversity in agroecosystems. Invertebrate Biodiversity as Bioindicators of Sustainable Landscapes.

[7] Zanin G., Otto S., Riello L., Borin M. (1997). Ecological interpretation of weed flora dynamics under different tillage systems. Agric. Ecosyst. Environ..

[8] Fracchiolla M., Caramia D., Lasorella C., Montemurro P. (2013). Ground cover management strategies in an Apulian oil-producing olive grove: Agronomic and ecological assessment proposals. Adv. Hortic. Sci..

[9] Fracchiolla M., Terzi M., Frabboni L., Caramia D., Lasorella C., De Giorgio D., Montemurro P., Cazzato E. (2015). Influence of different soil management practices on ground-flora vegetation in an almond orchard. Renew. Agric. Food Syst..

[10] MacLaren C., Bennett J., Dehnen-Schmutz K. (2019). Management practices influence the competitive potential of weed communities and their value to biodiversity in South African vineyards. Weed Res..

[11] Soriano M.A., Álvarez S., Landa B.B., Gómez J.A. (2014). Soil properties in organic olive orchards following different weed management in a rolling landscape of Andalusia, Spain. Renew. Agric. Food Syst..

[12] Martínez J.R.F., Zuazo V.H.D., Raya A.M. (2006). Environmental impact from mountainous olive orchards under different soil-management systems (SE Spain). Sci. Total Environ..

[13] Castro J., Fernández-Ondoño E., Rodríguez C., Lallena A.M., Sierra M., Aguilar J. (2008). Effects of different olive-grove management systems on the organic carbon and nitrogen content of the soil in Jaén (Spain). Soil Tillage Res..

[14] Gkisakis V.D., Kollaros D., Kabourakis E.M. (2014). Soil arthropod biodiversity in plain and hilly olive orchard agroecosystems, in Crete, Greece. Entomol. Hell..

[15] Simoes M.P., Belo A.F., Pinto-Cruz C., Pinheiro A.C. (2014). Natural vegetation management to conserve biodiversity and soil water in olive orchards. Span. J. Agric. Res..

[16] Calabrese G., Perrino E.V., Ladisa G., Aly A., Tesfmichael Solomon M., Mazdaric S., Benedetti A., Ceglie F.G. (2015). Short-term effects of different soil management practices on biodiversity and soil quality of Mediterranean ancient olive orchards. Org. Agric..

[17] Sánchez-Moreno S., Castro J., Alonso-Prados E., Alonso-Prados J.L., García-Baudín J.M., Talavera M., Durán-Zuazo V.H. (2015). Tillage and herbicide decrease soil biodiversity in olive orchards. Agron. Sustain. Dev..

[18] Carpio A.J., Castro J., Tortosa F.S. (2019). Arthropod biodiversity in olive groves under two soil management systems: Presence versus absence of herbaceous cover crop. Agric. For. Entomol..

[19] Carpio A.J., Lora Á., Martín-Consuegra E., Sánchez-Cuesta R., Tortosa F.S., Castro J. (2020). The influence of the soil management systems on aboveground and seed bank weed communities in olive orchards. Weed Biol. Manag..

[20] Carpio A.J., Oteros J., Tortosa F.S., Guerrero-Casado J. (2016). Land use and biodiversity patterns of the herpetofauna: The role of olive groves. Acta Oecologica.

[21] Cohen M., Bilodeau C., Alexandre F., Godron M., Andrieu J., Grésillon E., Garlatti F., Morganti A. (2015). What is the plant biodiversity in a cultural landscape? A comparative, multi-scale and interdisciplinary study in olive groves and vineyards (Mediterranean France). Agric. Ecosyst. Environ..

[22] Rey P.J. (2011). Preserving frugivorous birds in agro-ecosystems: Lessons from Spanish olive orchards. J. Appl. Ecol..

[23] Gonçalves M.F., Pereira J.A. (2012). Abundance and diversity of soil arthropods in the olive grove ecosystem. J. Insect Sci..

[24] Fleskens L., Duarte F., Eicher I. (2009). A conceptual framework for the assessment of multiple functions of agro-ecosystems: A case study of Trás-os-Montes olive groves. J. Rural Stud..

[25] Calabrese G., Tartaglini N., Ladisa G. (2012). Studio Sulla Biodiversità Negli Oliveti Secolari.

[26] Pe’Er G., Zinngrebe Y., Moreira F., Sirami C., Schindler S., Müller R., Bontzorlos V., Clough D., Bezák P., Bonn A. (2019). A greener path for the EU Common Agricultural Policy. Science.

[27] Barberi P., Burgio G., Dinelli G., Moonen A.C., Otto S., Vazzana C., Zanin G. (2010). Functional biodiversity in the agricultural landscape: Relationships between weeds and arthropod fauna. Weed Res..

[28] ISTAT (2010). http://censimentoagricoltura.istat.it/index.php?id=73.

[29] Brullo S., del Galdo G.G., Guarino R., Minissale P. (2007). A survey of the weedy communities of Sicily. Ann. Bot..

[30] Collavo A., Sattin M. (2011). Resistance to glyphosate in *Lolium rigidum* selected in Italian perennial crops: Bioevaluation, management and molecular bases of target-site resistance. Weed Res..

[31] Sansom M., Saborido A.A., Dubois M. (2013). Control of *Conyza* spp. with glyphosate-a review of the situation in Europe. Plant Protect. Sci..

[32] GIRE, Italian Herbicide Resistance Working Group (2020). Database of Herbicide Resistance in Italy. www.resistenzaerbicidi.it.

[33] Rodrigues M.A., Cabanas J., Lopes J., Pavão F., Aguiar C., Arrobas M. (2009). Ground cover and dynamic of weeds after the introduction of herbicides as soil management system in a rainfed olive orchard. Rev. Ciências Agrárias (Port.).

[34] Solomou A.D., Sfougaris A.I., Kalburtji K.L., Nanos G.D. (2013). Effects of organic farming on winter plant composition, cover and diversity in olive grove ecosystems in central Greece. Commun. Soil Sci. Plan..

[35] Oerke E.C. (2006). Crop losses to pests. J. Agric. Sci..

[36] Storkey J., Neve P. (2018). What good is weed diversity?. Weed Res..

[37] Norris R.F., Kogan M. (2000). Interactions between weeds, arthropod pests, and their natural enemies in managed ecosystems. Weed Sci..

[38] Norris R. (2005). Ecological bases of interactions between weeds and organisms in other pest categories. Weed Sci..

[39] Marshall E.J.P., Brown V.K., Boatman N.D., Lutman P.J.W., Squire G.R., Ward L.K. (2003). The role of weeds in supporting biological diversity within crop fields. Weed Res..

[40] Gerowitt B., Bertke E., Hespelt S.K., Tute C. (2003). Towards multifunctional agriculture—Weeds as ecological goods?. Weed Res..

[41] Swanton C.J., Weise S.F. (1991). Integrated weed management: The rationale and approach. Weed Technol..

[42] Cléments D.R., Weise S.F., Swanton C.J. (1994). Integrated weed management and weed species diversity. Phytoprotection.

[43] Harker K.N., O’Donovan J.T. (2013). Recent weed control, weed management, and integrated weed management. Weed Technol..

[44] Radić Lakoš T., Milović M.D., Jelaska S. (2014). Possible implications of two management types in olive groves on plant diversity. Agric. Conspec. Sci..

[45] Biondi E., Biscotti N., Casavecchia S., Marrese M. (2007). Oliveti secolari: Habitat nuovo proposto per l’inserimento nell’Allegato I della Direttiva (92/43 CEE). Fitosociologia.

[46] Perrino E.V., Ladisa G., Calabrese G. (2014). Flora and plant genetic resources of ancient olive groves of Apulia (Southern Italy). Genet. Resour. Crop. Evol..

[47] Perrino E.V., Wagensommer R.P., Medagli P. (2014). *Aegilops* (*Poaceae*) in Italy: Taxonomy, geographical distribution, ecology, vulnerability and conservation. Syst. Biodivers..

[48] Terzi M. (2015). Numerical analysis of the order *Scorzoneretalia villosae*. Phytocoenologia.

[49] Mucina L., Bültmann H., Dierßen K., Theurillat J.-P., Raus T., Čarni A., Šumberová K., Raus T., Di Pietro R., Gavilán R. (2016). Vegetation of Europe: Hierarchical floristic classification system of vascular plant, bryophyte, lichen, and algal communities. Appl. Veg. Sci..

[50] Gabriel D., Thies C., Tscharntke T. (2005). Local diversity of arable weeds increases with landscape complexity. Perspect. Plant Ecol. Evol..

[51] Russo D., Jones G. (2003). Use of foraging habitats by bats in a Mediterranean area determined by acoustic surveys: Conservation implications. Ecography.

[52] Davy C.M., Russo D., Fenton M.B. (2007). Use of native woodlands and traditional olive groves by foraging bats on a Mediterranean island: Consequences for conservation. J. Zool..

[53] Rey P.J., Manzaneda A.J., Valera F., Alcántara J.M., Tarifa R., Isla J., Molina-Pardoa J.L., Calvoa G., Salidoa T., Eugenio Gutiérrez J. (2019). Landscape-moderated biodiversity effects of ground herb cover in olive groves: Implications for regional biodiversity conservation. Agric. Ecosyst. Environ..

[54] Castro-Caro J.C., Barrio I.C., Tortosa F.S. (2014). Is the effect of farming practices on songbird communities landscape dependent? A case study of olive groves in southern Spain. J. Ornithol..

[55] Herrera J.M., Costa P., Medinas D., Marques J.T., Mira A. (2015). Community composition and activity of insectivorous bats in Mediterranean olive farms. Anim. Cons..

[56] Allen H.D., Randall R.E., Amable G.S., Devereux B.J. (2006). The impact of changing olive cultivation practices on the ground flora of olive groves in the Messara and Psiloritis regions, Crete, Greece. Land Degrad. Dev..

[57] Tscharntke T., Klein A.M., Kruess A., Steffan-Dewenter I., Thies C. (2005). Landscape perspectives on agricultural intensification and biodiversity–ecosystem service management. Ecol. Lett..

[58] Rivas-Martínez S., Penas Á., del Río S., González T.E.D., Rivas-Sáenz S., Loidi J. (2017). Bioclimatology of the Iberian Peninsula and the Balearic Islands. The Vegetation of the Iberian Peninsula.

[59] Westhoff V., Van Der Maarel E. (1978). The Braun-Blanquet approach. Classification of Plant Communities.

[60] Chytrý M., Otýpková Z. (2003). Plot sizes used for phytosociological sampling of European vegetation. J. Veg. Sci..

[61] Braun-Blanquet J., Fuller G.D., Conrad G.D. (1932). Plant Sociology.

[62] Lepš J., Hadincová V. (1992). How reliable are our vegetation analyses?. J. Veg. Sci..

[63] Pignatti S., Menegoni P., Pietrosanti S. (2005). Biondicazione attraverso le piante vascolari. Valori di indicazione secondo Ellenberg (Zeigerwerte) per le specie della Flora d’Italia. Braun-Blanquetia.

[64] Euro+Med, 2006–2020. Euro+Med PlantBase—The Information Resource for Euro-Mediterranean Plant Diversity. http://ww2.bgbm.org/EuroPlusMed/.

[65] McCune B., Grace J.B. (2002). Analysis of Ecological Communities.

[66] Conti F., Manzi A., Pedrotti F. (1997). Liste Rosse Regionali Delle Piante d’Italia.

[67] Wagensommer R.P., Medagli P., Perrino E.V. (2013). Piante vascolari minacciate e Liste Rosse: Aggiornamento delle conoscenze in Puglia. Inform. Bot. Ital..

[68] Conti F., Manzi A., Pedrotti F. (1992). Libro Rosso Delle Piante d’Italia.

[69] Orsenigo S., Fenu G., Gargano D., Montagnani C., Abeli T., Alessandrini A., Bacchetta G., Bartolucci F., Carta A., Castello M. (2021). Red list of threatened vascular plants in Italy. Plant Biosyst..

[70] Bilz M., Kell S.P., Maxted N., Lansdown R.V. (2011). European Red List of Vascular Plants.

[71] Mielke P.W., Berry K.J. (2001). Permutation Methods.

[72] McCune B., Mefford M.J. (2011). PC-ORD. Multivariate Analysis of Ecological Data.

[73] Brooks M.E., Kristensen K., van Benthem K.J., Magnusson A., Berg C.W., Nielsen A., Skaug H.J., Maechler M., Bolker B.M. (2017). glmmTMB balances speed and flexibility among packages for zero-inflated generalized linear mixed modeling. R J..

[74] Dufrêne M., Legendre P. (1997). Species assemblages and indicator species: The need for a flexible asymmetrical approach. Ecol. Monogr..

[75] De Cáceres M., Legendre P., Moretti M. (2010). Improving indicator species analysis by combining groups of sites. Oikos.

[76] Wagensommer R.P., Medagli P., Turco A., Perrino E.V. (2020). IUCN Red List evaluation of the Orchidaceae endemic to Apulia (Italy) and considerations on the application of the IUCN protocol to rare species. Nat. Conserv. Res..

